# Editorial: Ensuring Animal Health and Other Services for Efficient and Inclusive Livestock Value Chains in LMICs

**DOI:** 10.3389/fvets.2021.807516

**Published:** 2021-12-14

**Authors:** Isabelle Baltenweck, Simeon Kaitibie, Bernard Bett

**Affiliations:** ^1^Policies Institutions and Livelihoods Program, International Livestock Research Institute (ILRI), Nairobi, Kenya; ^2^Animal and Human Health Program, Faculty of Agribusiness and Commerce, Lincoln University, Lincoln, New Zealand

**Keywords:** value chain, low and medium income countries, livelihoods, delivery system, livestock, animal health

The livestock sector offers opportunity for many livestock producers in LMICs to improve their livelihoods (1). Under various scenarios (2), the demand for livestock-derived foods will continue to increase in these countries, offering market incentives to increase livestock production and productivity. However, livestock productivity remains very low. For example, annual milk yield of a cow in Sub-Saharan Africa and South Asia is 6 and 12%, respectively, of a cow in an OECD country. Within countries and production systems, yield gaps are high for all species (3). This suggests that productivity increase is feasible with changes in management, breeds, feeds and health practices, also considering ecological and social economic factors. One of the key constraints faced by livestock keepers is access to affordable and quality inputs and services—all needed to improve productivity. These include animal health inputs and services, feed and breeding, and also extension or advisory services. Different organizational arrangements for the delivery of such inputs and services have emerged, especially in the dairy and poultry sectors. Some of these arrangements are led by value chain actors themselves, while others have been promoted and supported by development agencies and donors. The effectiveness of these organizational arrangements remains insufficiently documented, limiting the opportunity to learn and apply lessons across value chains and countries. The objective of the research topic is to facilitate research and stimulate discussion regarding access to affordable and quality inputs and services that ultimately improve livestock production and productivity in a sustainable and equitable way.

The 12 papers included in this research topic cover a range of topics- nine papers focus on animal inputs and services (including seven on animal health, two on breeding/genetics and one on extension); one paper covers both health and genetics. In addition, two papers are about output markets and one on policies. Seven of the 12 papers are outputs of the CGIAR Research Program on Livestock that “aims to create a well-nourished, equitable, and environmentally healthy world through livestock research for development.” The 12 submissions follow three general methodologies.

The first set of papers falls into the “characterization studies” category. Three papers deal with the provision of animal health inputs and services. They highlight the inadequacy between livestock keepers' demand for these services, and the supply. Enahoro et al. presents a clear example of this phenomenon in the case of the poultry sector in Ghana and Tanzania. In addition, Gizaw et al. describes the co-existence of formal and informal systems in the provision of animal health services in Ethiopia and the dissatisfaction with the public sector in reaching particularly the pastoral community. Authors in this first set of papers also highlight the need for increased capacity development of service providers. This is exemplified with respect to food safety considerations in Dione et al. in the case of use of antimicrobial drugs in Uganda, and Murungi et al. in the case of pig traders and abattoirs in Nairobi, Kenya. The challenge of limited access and availability and low quality of inputs and services was also highlighted in the policy review of the dairy sector in Rwanda by Habiyaremye et al.

The second set of papers uses ex ante impact evaluation methodologies, to assess the likely effects of specific interventions in guiding policies and investments. One paper (Ouma et al.) focuses on farmers' demand for a vaccine against pig cysticercosis in Uganda; it concludes that as markets may not recognize this public health cost, pig producers will be willing to pay for vaccine only if they can pass on the costs to consumers. The authors call for public health interventions as private delivery of such a vaccine will likely not be successful. Also, to guide policies while focusing on output, Rich and Wane analyze the option for the cattle sector in Burkina Faso to shift from exporting beef (with the setup of abattoirs) in lieu of live animals to Ghana. Given the low competitiveness of the West African meat, compared to other imports, the authors urge that focus should rather be on increasing livestock productivity.

The third set of papers looks at the effects of new or improved livestock services, including their delivery on households' livelihoods. Dione et al. assesses the change in knowledge amongst pig producers in Uganda following the introduction of extension services using Interactive Voice Response (IVR) technology in delivering biosecurity messages for the control of African swine fever (ASF). Their study shows positive results in terms of improved knowledge, for those who had not had any training earlier but also to enhance knowledge for these who attended face to face trainings. Two other papers using this methodology analyze change in livestock productivity and income: Kassie et al. in the case of delivery of breeding and health services for small ruminants in Ethiopia and Teufel et al. for the case of the Infection and Treatment Method (ITM) against East Coast Fever in Tanzania. Kassie et al. using different specifications of difference-in-differences models show that access to small ruminant health services has increased a range of livestock productivity indicators (offtake, return per head, and gross income per adult equivalent). A similar conclusion was reached when comparing users of the ITM technology and the non-users (or rather these who adopted recently) in Tanzania. These two studies highlight the potential for livestock innovations to have positive and long-lasting effects on livelihoods.

It is also worth noting that a couple of studies highlighted gender differences. Among them, Gizaw et al. shows that women have lower access to animal health services in general in Ethiopia while Enahoro et al. makes the same observation for poultry farmers in Ghana and Tanzania. Extension services using mobile technology like IVR show less gender differences, as noted by Dione et al. in the case of pig farming in Uganda.

Overall, this Research Topic provides a good overview of the situation and challenges with respect to the delivery of livestock inputs and services, with a focus on Sub Saharan Africa. The papers discuss in particular the role of the public and private sectors, and the importance of unlicensed, informal, service providers. Interestingly no paper covered producer organizations as institutions supporting livestock producers' access to inputs and services, despite some evidence of their importance. It is also worth noting that many papers are characterization studies, with only three providing much-needed assessment of the effects of innovations, or new ways to provide inputs and services—on livestock productivity and resulting households' livelihoods. These three studies show that rigorous research design, while complex, is feasible, and the results are key in guiding further investments for the livestock sector in LMICs.

## Author Contributions

IB drafted the paper. SK and BB revised the manuscript. All authors contributed to the article and approved the submitted version.

## Conflict of Interest

The authors declare that the research was conducted in the absence of any commercial or financial relationships that could be construed as a potential conflict of interest.

## Publisher's Note

All claims expressed in this article are solely those of the authors and do not necessarily represent those of their affiliated organizations, or those of the publisher, the editors and the reviewers. Any product that may be evaluated in this article, or claim that may be made by its manufacturer, is not guaranteed or endorsed by the publisher.
